# B lymphocyte stimulator, interferon-α and HMGB 1 interrelation in childhood onset systemic lupus erythematosus: associations with disease activity and severity

**DOI:** 10.1186/1546-0096-12-S1-P321

**Published:** 2014-09-17

**Authors:** Florence Kanakoudi-Tsakalidou, Vasiliki Tzimouli, Evangelia Farmaki, Maria Trachana, Anna Taparkou, Panagiota Nalbanti, Polyxeni Pratsidou-Gertsi, Fotis Papachristou

**Affiliations:** 1 1st Dept of Pediatrics, Aristotle University, Pediatric Immunology and Rheumatology Referral Center, Ippokration Hospital, Thessaloniki, Greece

## Introduction

The role of B lymphocyte stimulator (Blys) in Childhood onset Systemic Lupus Erythematosus (cSLE) has not been elucidated.

## Objectives

To investigate the role of Blys and its association with biomarkers related with the cSLE disease activity and severity.

## Methods

34 cSLE patients (study group), 51 with other rheumatic or autoimmune diseases and 26 healthy children (control groups) were studied. For evaluating the disease activity, SLEDAI and ECLAM scores were used. For estimating the disease severity the criteria were: involvement of 3 vital organs (kidneys or CNS or blood or combination of ≥2 organs), increased anti-dsDNA titres, low C3 /C4 concentrations and high concentrations of serum IFN-α and HMGB1 (High mobility group box 1) protein.

## Results

Mean Blys levels were significantly higher in cSLE patients in respect to patients of all control groups, in patients with active as compared with patients with inactive disease and in patients with vital organ involvement as compared with patients without such involvement. Moreover, mean Blys levels were positively correlated with anti-DNA titer, IFN-α and HMGB1 levels but negatively correlated with C3 /C4 concentrations.

## Conclusion

Taking together, results of our study indicate that: high levels of Blys are mainly found in serum of jSLE patients and they may be involved in the increased anti-DNA plus other autoantibody production. Moreover, they are associated with the disease activity and severity, meaning that Blys is a specific biomarker for the selection of patients who will need more aggressive and possibly more targeted than conventional therapy.

## Disclosure of interest

None declared

